# Language-like efficiency and structure in house finch song

**DOI:** 10.1098/rspb.2024.0250

**Published:** 2024-04-03

**Authors:** Mason Youngblood

**Affiliations:** ^1^ Minds and Traditions Research Group, Max Planck Institute for Geoanthropology, Jena, Thüringen, Germany; ^2^ Institute for Advanced Computational Science, Stony Brook University, Stony Brook, NY, USA

**Keywords:** birdsong, communicative efficiency, linguistic laws, hierarchical structure, cultural evolution

## Abstract

Communication needs to be complex enough to be functional while minimizing learning and production costs. Recent work suggests that the vocalizations and gestures of some songbirds, cetaceans and great apes may conform to linguistic laws that reflect this trade-off between efficiency and complexity. In studies of non-human communication, though, clustering signals into types cannot be done *a priori*, and decisions about the appropriate grain of analysis may affect statistical signals in the data. The aim of this study was to assess the evidence for language-like efficiency and structure in house finch (*Haemorhous mexicanus*) song across three levels of granularity in syllable clustering. The results show strong evidence for Zipf's rank–frequency law, Zipf's law of abbreviation and Menzerath's law. Additional analyses show that house finch songs have small-world structure, thought to reflect systematic structure in syntax, and the mutual information decay of sequences is consistent with a combination of Markovian and hierarchical processes. These statistical patterns are robust across three levels of granularity in syllable clustering, pointing to a limited form of scale invariance. In sum, it appears that house finch song has been shaped by pressure for efficiency, possibly to offset the costs of female preferences for complexity.

## 1. Introduction

### (a) Efficiency and complexity

Communication systems tend to be optimized for efficiency, or the benefit that they bestow relative to the costs of learning and producing them:

1.1 $$\mathrm{efficiency}\propto \frac{\sum \pi }{C_{L}+\sum C_{P}},$$

where ∑π is the lifetime benefit of the communication system (which includes perception by receivers), $C_{L}$ is the cost of learning it, and $\sum C_{P}$ is the lifetime cost of producing it [1]. This framing is consistent with understandings of efficiency in linguistics [2]: the famous ‘principle of least effort’ can be thought of as a minimization of these costs [3].

On the other hand, simpler sounds that are easier to learn and produce vary across fewer dimensions and are less distinctive from one another [4]. Signals need to be distinguishable to be functional [5], and complexity expands the possibilities within a signal space in a way that enhances functionality (e.g. to communicate concepts in language, or attract mates in birdsong) [6]. But there is, of course, a limit. Simulations show that cultural evolution plateaus when complex behaviors are too costly [7]. Evidence from linguistics, animal behaviour and cultural evolution suggests that communication systems generally evolve to balance this complexity–efficiency trade-off [1,2,8].

Notions of complexity can vary quite widely. In the birdsong literature, complexity is usually described at one of three levels: syllables (individual sounds within songs), songs (sequences of syllables) or repertoires (full set of syllables or songs that a bird produces). Syllable and repertoire complexity are approximated with measures of production cost (e.g. frequency bandwidth, number of transitions in pitch [9]) and learning cost (e.g. diversity of unique syllables or songs [10]), respectively. Song complexity, on the other hand, is often characterized by measures of hierarchical or combinatorial structure [11,12]. Structured signals are more compressible and learnable [2,13,14], which allows more information to ‘pass through the bottleneck’ of cultural transmission [1,15]. Syllable complexity increases both production and learning costs, whereas song complexity decreases learning costs. Both notions of complexity, however, should boost the benefits of communication by expanding the signal space. Syllable complexity allows for greater diversity in syllable types, and song complexity allows those syllable types to be combined into a wider array of song types.

The effect of this complexity–efficiency trade-off has been confirmed by experiments showing that artificial languages gain efficiency [16] and structure [17] as they are culturally transmitted and used by participants. Studies of continuous whistled communication systems that resemble birdsong have also detected increases in combinatorial structure (i.e. signals comprised constituent parts that are recombined in different ways) [14]. Birdsong experiments yield similar results. Zebra finches raised in isolation sing atypical songs, but over several generations they converge towards wild-type song by shortening the longest syllables and gaining spectral structure [18]. Even foraging behaviors that are culturally transmitted become more efficient over time, a tendency that is accelerated by population turnover because new group members are more likely to adopt the more efficient behavior [19].

### (b) Linguistic laws and structure

Pressure for compression and efficiency lead to regularities in organization that are so universal in human language that they are referred to as linguistic laws. The three most commonly studied of these are Zipf's rank–frequency law, Zipf's law of abbreviation and Menzerath's law [20].

Zipf's rank–frequency law predicts that the frequency of an item will be proportional to the inverse of its rank (i.e. first most common, second most common, etc.), a relationship that holds for most, if not all, of the world's languages [21]. In this study, I will focus on Mandelbrot's more flexible parameterization of Zipf's rank–frequency law (see Analysis) [22,23], which is its most common form in contemporary linguistics [21]. Non-human animal communication systems, including bird and cetacean vocalizations [24–28] and lizard courtship displays [29], exhibit more redundancy than languages, leading to a convex rank–frequency relationship that is better captured by the Zipf–Mandelbrot distribution. Zipf and Mandelbrot both interpreted this rank–frequency law as resulting from a minimization of production and perception costs [3,22], and there are models showing that it can be derived from communicative efficiency [30–32]. However, some of these models assume that signals map to objects or concepts which is not the case in birdsong, and other causes are still debated [21]. Even though there is still uncertainty about its causes, the presence of Zipf's rank–frequency law in non-human communication systems has been interpreted as evidence for both communicative efficiency and information content [33,34].

Zipf's law of abbreviation predicts that common items will tend to be shorter than rare items because their production cost is lower [3]. This negative correlation between frequency and duration is widespread in both written [35] and spoken language [36], and has also been observed in writing systems [37] and non-human communication like chimpanzee gestures [38] and bird and primate vocalizations [39–41]. The explanation for Zipf's law of abbreviation is simple: when common items have a lower production cost than rare items then the overall production cost of signals goes down [42]. Shorter signals carry other benefits as well, such as reduced predation risk and reverberation in the environment [42].

Menzerath's law predicts that longer sequences will be comprised shorter items to balance production costs [43]. This negative correlation between sequence length and item length is found at various levels of analysis in language (e.g. clauses in sentences, morphemes in words) [44–46]. In non-human communication, Menzerath's law appears to be present in chimpanzee gesture [38] and in the vocalizations of some primates and birds [40,41,47,48], including house finches [49]. The explanation for Menzerath's law is an extension of Zipf's law of abbreviation: when production costs are increased in one domain (e.g. song sequence length) they should be decreased in another (e.g. syllable duration).

Beyond linguistic laws, there are two proxy measure of linguistic structure that have recently been investigated in non-human communication: small-world structure [50] and mutual information decay [12].

Small-world networks are highly clustered and have short average path lengths, so that it only takes a few steps to jump between any pair of nodes (think ‘six degrees of separation’) [50,51]. These sorts of networks are quite common in biological and social systems [50,51], including language. For example, networks of neighbouring words in sentences and co-occurring words in thesauruses exhibit small-world structure [52,53]. Small-worldness is thought to reflect general systematic structure and recurrence, which in turn improve the compressibility and learnability of information [13,24,54]. It is also hypothesized to reflect the emergence of syntactic structure over time [55], as both nightingales and children in more advanced stages of vocal development have greater small-world structure in their transition networks [56,57]. Humpback whales [24] and several songbird species [26,56,58,59] all exhibit small-worldness in their song syntax to a similar degree.

Mutual information is a measure of dependency, or the amount of information that the presence of one thing has about another. In the context of language, past words provide information that can help predict future words above chance levels (i.e. ‘do you need anything from the’ → ‘store’). Intuitively, a word contains more information about the very next word than the one that comes after it [60], but correlations can be detected even at very long distances of hundreds of words [61]. The rate at which the mutual information of words decreases with increasing distance can provide clues about underlying syntactic structure. Simple models of grammar that assume Markov processes (i.e. next word depends only on last few words) lead to exponential decay in mutual information with distance, whereas hierarchical processes (i.e. word order comes from nested syntactic rules that allow for long-range dependencies [62], a common view going back to Chomsky [63]) lead to power-law decay [64,65]. In German, Italian, Japanese and English, mutual information decay is exponential at short distances and fits a power-law at long distances, suggesting that sequences are generated by a combination of Markovian and hierarchical processes [12,66]. This pattern has also been documented in four bird species, suggesting that sequential organization of song is more complex than previously thought [12].

### (c) Granularity

**Figure 1.**
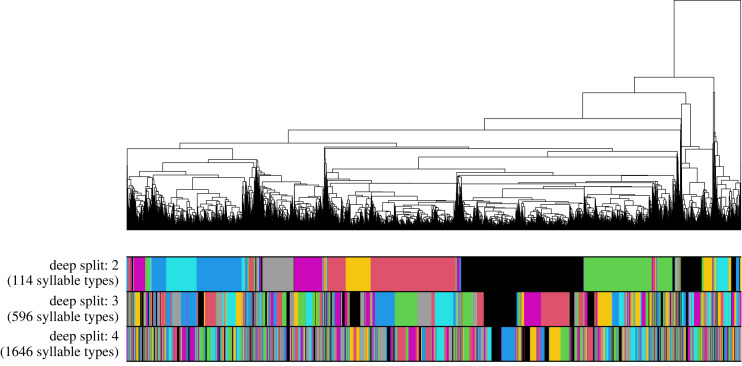
The results of hierarchical clustering. The coloured bars below the dendrogram correspond to the categories assigned to each syllable when deep split is 2 (over-lumping), 3 (baseline) and 4 (over-splitting).

In languages, the boundaries between signals are apparent to the humans who use them. In non-human communication systems, categorizing signals into types is its own challenge. For relatively small datasets it is possible to manually inspect recorded signals and assign them into types. Increasingly, researchers are turning to automated methods such as hierarchical clustering, dimension reduction, and machine learning that classify signals into types based on their acoustic features. Automated methods that classify signals into types based on their acoustic features [67–69] reduce subjectivity and enable people to work with much more data, but they also require tuning. For example, hierarchical clustering requires the user to choose an appropriate threshold below which the ‘branches’ of the ‘tree’ are combined into types (figure 1). This threshold effectively controls the granularity of clustering: higher values over-lump signals into fewer categories, while lower values over-split signals into more categories. The granularity of an analysis may influence the kinds of patterns that can detected. Philosophers of cultural evolution call this the ‘grain problem’—some statistical patterns may be more apparent at certain levels of analysis [70].

### (d) Aim and model

The aim of this study is to assess the evidence for language-like efficiency and structure in house finch (*Haemorhous mexicanus*) song across three levels of granularity in syllable clustering. By doing so, I hope to (1) identify which features of birdsong may be most subject to the complexity–efficiency trade-off, and (2) determine how clustering decisions affect the manifestation of linguistic laws in non-human communication systems. The data for this study come from a large corpus of house finch songs collected between 1975 and 2019 [9,71,72]. House finch song is an excellent model for these questions for several reasons. First, house finch song is socially learned [73] and culturally evolves [71], and thus should be subject to information compression. Second, male house finches are more likely to learn complex syllables [9]—a content bias that may be an adaptation to female preferences for complexity. In house finches, males that sing longer songs at a faster rate are more attractive [74] and have higher reproductive performance [75], and courtship songs are longer and contain more syllable types [76]. Because these measures of complexity relate to production and learning costs, they may increase pressure for efficiency in other domains such as duration. Finally, house finch song is known to be subject to efficiency constraints. When house finches tutored by canaries reproduce the trills of their foster parents they are slower and much shorter [73], and house finches increase the frequency of their vocalizations to minimize competition with the lower frequency sounds [77].

## 2. Analysis

Unless otherwise stated, all models were fit in STAN using the brms package in R [78], with 20 000 iterations across four MCMC chains. Prior specifications, model diagnostics and full model output for all analyses can be found in the electronic supplementary material.

### (a) Data

The recordings used in this study (2724 songs from 331 individuals) were collected in 1975 [71], 2012 [72] and 2019 [9] in the New York metropolitan area, and analysed by Youngblood & Lahti [9] using Luscinia (https://rflachlan.github.io/Luscinia/) (full recording and analysis details, and an example of an analysed song, are in Youngblood & Lahti [9] and the electronic supplementary material). In this population, males have a repertoire of approximately 35–40 syllable types, which they combine into 1–7 stereotyped song types (mean of 3.1) that are between 6–31 syllables long (mean of 12.1) [72]. The main analysis was conducted using recordings from all three years, but the patterns are qualitatively the same when each year is analysed separately (see electronic supplementary material).

### (b) Clustering

Clustering was conducted using the log-transformed mean frequency traces (mean frequency in each 1 ms bin) for each syllable in every song [79]. I used the mean frequency traces for clustering because they are time series, which means they capture more subtle variation than other features from Luscinia (e.g. overall bandwidth) and can be compared using dynamic time warping. First, the normalized distances between all of the syllables were calculated via dynamic time warping with a window size of 10 (10% of the average signal length) using the dtwclust package in R [80]. A window size of 10% of the signal length is commonly used in speech processing research and seems to be a practical upper limit for many applications [81]. Infinite distances (0.19% of cases) caused by comparisons of syllables with extreme signal length differences were assigned the maximum observed distance value. Next, hierarchical clustering and dynamic tree cut were used to cluster the syllables into types [82]. Hierarchical clustering was conducted with the UPGMA method implemented in the fastcluster package in R [83], and dynamic tree cut was conducted with the dynamicTreeCut package in R [84].

For dynamic tree cut, I ran the hybrid algorithm with a minimum cluster size of 1 to maximize the representation of rare syllable types, and used the deep split parameter (*DS*) to determine the granularity of clustering (details of *DS* are in the electronic supplementary material). I restricted this analysis to *DS* = {2,3,4} because values of *DS* = {0,1} lead to extremely unrealistic underestimates of the number of syllables types. *DS* = 2 leads to over-lumping of syllables into types (*n* = 114; low granularity), *DS* = 3 leads to a typical syllable classification (*n* = 596) [9,72,82], and *DS* = 4 lead to over-splitting (*n* = 1646; high granularity). The dendrogram and syllable classifications can be seen in figure 1. Unseen species models indicate that the sample of syllable types in the population is likely complete (see electronic supplementary material).

Automated clustering methods carry a risk of duration-bias—variation may be more detectable in longer syllables [85]. This risk should be minimized by the use of dynamic time warping and dynamic tree cut, but a replication of Zipf's law of abbreviation with manually classified syllables [73], as well as an exploratory analysis of duration bias in the automated clustering algorithm, can be found in the electronic supplementary material.

Once syllable types were identified by hierarchical clustering, I followed Ju *et al.* [72] and Youngblood & Lahti [9] in calculating the following syllable-level acoustic features for further analysis: average frequency (Hz), minimum frequency (Hz), maximum frequency (Hz), bandwidth (Hz), duration (ms), concavity (changes in the sign of the slope of the mean frequency trace/ms) and excursion (cumulative absolute change in Hz/ms). Concavity and excursion are both indicators of syllable complexity [72,86]. Concavity was calculated after smoothing the mean frequency trace using a polynomial spline with a smoothing parameter of 5 [9].

### (c) Zipf's rank–frequency law

Mandelbrot's generalization of Zipf's rank–frequency law takes the following form [22,23]:

2.1 $$f(r)=\frac{c}{{\left(r+\beta \right)}^{\alpha }},$$

where f(r) is the normalized frequency at each rank *r*, *c* is a normalization parameter, and α and β are parameters that control slope and convexity (respectively). According to Izsák [87], the bounds of (2) are α>1, β>−1, and c>1. When β=0, this function simplifies to the original form of Zipf's rank–frequency law: $f(r)\propto 1/r^{\alpha }$.

*c* is usually a normalization term defined as

2.2 $$c=\sum_{i=1}^{\infty }\frac{1}{{\left(r+\beta \right)}^{\alpha }}.$$

In practice, this form of Zipf's rank-frequency law is notoriously difficult to fit to data due to strong correlations between *α* and *β*, which in turn determine *c* [87]. Here, I use a simplified version of (2.1) that treats *c* as a third parameter that is estimated alongside *α* and *β* [88], as has been done in studies of chickadee calls [89,90], which should be interpreted as an approximation of the Zipf-Mandelbrot distribution.

I fitted (2.1) to the rank–frequency distributions of the syllable classifications from each level of deep split in two batches. First, I fitted all three parameters to approximate Mandelbrot's versions of the rank–frequency law. Then, I set β=0 to approximate Zipf's original formulation. The model was fitted as non-linear model on the original scale, as opposed to the log-log scale.

As there is no established hypothesis test for Zipf's rank–frequency law, I will simply report the goodness-of-fit (the norm in linguistics) and refer to it as ‘consistent’ with the law when it is within the range reported for human languages (*R*^2^ > 0.8) [21,91].

**Figure 2.**
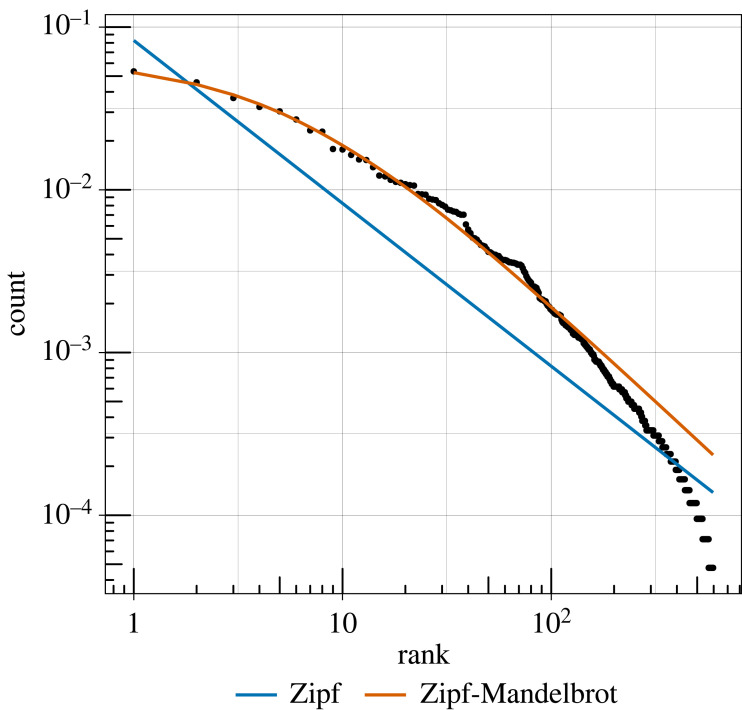
The relationship between rank (*x*-axis) and count (*y*-axis) at the intermediate granularity level (DS = 3, 596 syllable types). The blue and orange lines denote the expected distributions according to Zipf's rank–frequency law (blue; *a* = 1.00, *c* = 0.08) and Mandelbrot's extension of it (orange; *a* = 1.20, *b* = 5.69, *c* = 0.52).

The observed frequency distribution is consistent with the Zipf–Mandelbrot distribution at all three levels of granularity in syllable clustering ($R^{2}=\{0.995,0.995,0.995\}$ at DS={2,3,4}), but is inconsistent with the the original form of Zipf's rank frequency law at the two of the three levels of granularity ($R^{2}=\{0.945,0.777,0.523\}$). Figure 2 shows the rank–frequency distribution at the intermediate granularity level (DS=3; 596 syllable types), while all three can be seen in the electronic supplementary material. The distribution has a poorer fit to the rarer syllable types—a common pattern in human language [21] that is further investigated in the electronic supplementary material. The Zipf–Mandelbrot distribution also outcompetes Zipf's original form and has $R^{2}>0.98$ when models are fitted separately to the data from each year (1975, 2012 and 2019; see electronic supplementary material).

### (d) Zipf's law of abbreviation

Zipf's law of abbreviation predicts that common items will be shorter in duration than rarer ones. Rather than focusing duration alone, I explored whether the frequency of syllables is negatively correlated with four different measures of production cost: duration (ms), bandwidth (Hz), concavity (changes in the sign of the slope of the mean frequency trace/ms) and excursion (cumulative absolute change in Hz/ms).

For each level of deep split and each measure of production cost, I constructed a lognormal model with the measure in question as the outcome variable, count as the predictor variable, and syllable type as a varying intercept. The alternative formulation, a Poisson model with count as the outcome variable, does not allow for the correct random effects structure (e.g. counts are identical across observations of the same syllable type).

Duration, bandwidth, and excursion had strong negative effects on count at all three levels of granularity in syllable clustering (table 1). Concavity had no effect on count at the first two levels of granularity, but had a very weak negative effect at the third level of granularity. These patterns are apparent in the plots of production costs and counts in the electronic supplementary material. The results are qualitatively identical when the year of recording (1975, 2012 or 2019) is included as a varying intercept (see electronic supplementary material). Several other robustness checks, including replications of this analysis using the rank-based method from the R package ZLAvian [85,92] and using manually classified syllables from an experimental study in house finches [73], can be found in the electronic supplementary material.

**Table 1.** The estimated effect of count on each measure of production cost, using the syllable classifications from each level of deep split. 95% credible intervals that do not overlap with 0 are marked with an asterisk.

model	DS	Est.	Err.	2.5%	97.5%	
duration∼count	2	−0.36	0.14	−0.63	−0.10	*
	3	−0.46	0.06	−0.58	−0.34	*
	4	−0.42	0.03	−0.48	−0.35	*
bandwidth∼count	2	−0.55	0.11	−0.75	−0.34	*
	3	−0.68	0.06	−0.79	−0.57	*
	4	−0.64	0.03	−0.69	−0.58	*
concavity∼count	2	−0.02	0.10	−0.21	0.17	
	3	−0.06	0.04	−0.15	0.02	
	4	−0.07	0.03	−0.12	−0.02	*
excursion∼count	2	−0.26	0.07	−0.41	−0.11	*
	3	−0.33	0.04	−0.41	−0.25	*
	4	−0.32	0.02	−0.36	−0.27	*

### (e) Menzerath's Law

Menzerath's law predicts that longer sequences will be comprised smaller items. Importantly, Menzerath's law is sometimes detected in random sequences from null models [93–95]. There are two sorts of null models that make sense in this context: (1) random sequences with the same number of syllables as real songs, and (2) pseudorandom sequences that match the cumulative duration in time but can vary in the number of syllables. James *et al.* [49] interpret the latter as approximating simple motor constraints—Menzerath's law resulting from efficiency in production alone—where stronger effects in the real data would indicate additional mechanisms (e.g. communicative efficiency). In this study, I compare the real data against each of these to assess both the presence of Menzerath's law and whether it is beyond what would be expected from production constraints.

The production constraint model of James *et al.* [49] works as follows. For each iteration of the model, a pseudorandom sequence was produced for each real song in the dataset. Syllables were randomly sampled (with replacement) from the population until the duration of the random sequence exceeded the duration of the real song. If the difference between the duration of the random sequence and the real song was less than 50% of the duration of the final syllable, then the final syllable was kept in the sequence. Otherwise, it was removed. Each iteration of the model produces a set of random sequences with approximately the same distribution of durations as the real data.

To estimate the strength of Menzerath's law, I constructed a lognormal model with syllable duration as the outcome variable, song length in number of syllables as the predictor variable, and the song as a varying intercept [96]. The production constraint model removes variation accounted for by the year and individual bird, so song is the only varying intercept that is appropriate for comparison. This model was used to estimate the strength of the effect of song length on syllable duration in both the real data and 10 simulated datasets from both null models. The brm_multiple function from the brms package in R was used to fit a single model to the the 10 simulated datasets from each null model and produce a combined posterior distribution [78].

This analysis differs from James *et al.* [49] in two ways: (1) I use the actual duration of syllables rather than a single median value for each song, and (2) I compare the full posterior distributions rather than point estimates of effects. These decisions should yield more conservative conclusions. Note that syllable type is not incorporated into the modelling, so this is the only analysis that is not conducted across multiple levels of deep split.

**Figure 3.**
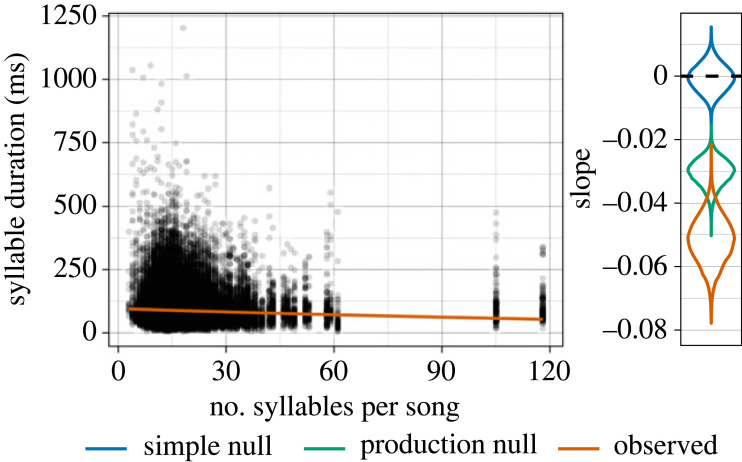
The relationship between song length in number of syllables (*x*-axis) and syllable duration (*y*-axis), with a best fit line from the fitted lognormal model. To the right of the main graph is the posterior distribution of the effect of song length on duration for the real data (orange) compared to 10 simulated datasets from the simple (blue) and production (green) null models. The combined posteriors for the simulated datasets are based on 2500 posterior samples from each of the 10 models.

The results of the lognormal model indicate that song length has a negative effect on syllable duration (mean estimate: −0.052; 95% CI: [−0.066, −0.038]) (left panel of figure 3). The posterior distribution for this effect from the model fit to the actual data (orange) is more negative than the posterior distributions from 10 simulated datasets from the production constraint null model of James *et al.* [49] (green) and a simple random null model (blue), although there is notable overlap between the lower tail of the production constraint model and the upper tail of the actual data (3.5% of the combined distribution area; right panel of figure 3). The negative effect of song length on syllable duration persists when the year of recording (1975, 2012 or 2019) is included as a varying intercept (see electronic supplementary material).

### (f) Small-worldness index

The small-worldness index (SWI) is a ratio based on the clustering coefficient (C) and average path length (L) [51]:

2.3 $$\mathrm{SWI}=\frac{C/C_{\mathrm{rand}}}{L/L_{\mathrm{rand}}},$$

where $C_{\mathrm{rand}}$ and $L_{\mathrm{rand}}$ are calculated from random networks of the same size as the real network. Values of SWI>1 are consistent with small-world structure [51], which is thought to reflect general systematic structure and thus compressibility [13,24].

**Figure 4.**
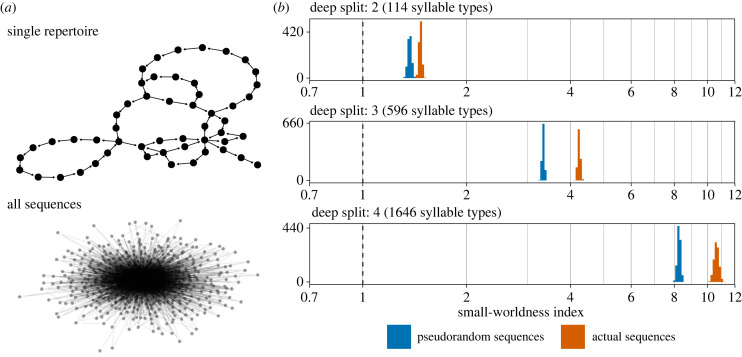
(*a*) Transition network between syllables types for a single male (#22 from 1975) above the global transition network for the entire dataset. (*b*) Estimated small-worldness index calculated 1000 times from the real data (orange) compared to pseudorandom sequences with the same frequency distribution of syllable types (blue). The dashed vertical line at 1 corresponds to the standard threshold for a network having small-world structure [51].

In this study, I followed Allen *et al.* [24] in calculating SWI from the unweighted directed network of all syllable transitions in the population (lower panel of figure 4*a*) to allow comparison with previous studies of non-human song. Importantly, if the frequency distribution of syllable types is very skewed, then random sequences could exhibit small-world structure simply because of clustering around common types. To avoid this confound, SWI was calculated 1000 times from the real data and 1000 times from random sequences with the same distribution of types.

Figure 4*b* shows the distribution of SWI calculated from random sequences with the same distribution of types (orange) and the actual sequences (blue). At all three levels of granularity in syllable clustering, the observed small-worldness of the real songs is above both the standard threshold (SWI>1) and the pseudorandom sequences. SWI>1 when computed separately from the data from each year (1975, 2012, and 2019; see electronic supplementary material)

### (g) Mutual information

The rate at which the mutual information between items decays with distance reflects whether underlying syntactic structure is Markovian, hierarchical or a composite of the two [12]. Mutual information (MI) was calculated from pairs of sequential syllables separated by a certain distance, using the method of Sainburg *et al.* [12]. MI was computed on concatenated sequences of songs from each individual. For example, if there are two individuals with two concatenated sequences of songs, a→b→c→d and e→f→g, and the target distance is 2, then the pairs used for MI calculated would be ((a,c),(b,d),(e,g)). MI would then be calculated using:

2.4 $$\hat{I}(X,Y)=\hat{S}(X)+\hat{S}(Y)-\hat{S}(X,Y),$$

where $\hat{I}$ denotes information and $\hat{S}$ denotes entropy. *X* is the distribution of first syllables (a,b,e), *Y* is the distribution of second syllables (c,d,g), and XY is the joint distribution of pairs ((a,c),(b,d),(e,g)). $\hat{S}$ was calculated using the method of Grassberger [97,98] used by Lin and Tegmark [65]:

2.5 $$\hat{S}=ln(N)-\frac{1}{N}\sum_{i=1}^{K}N_{i}\psi (N_{i}),$$

where *N* is the total number of tokens in the distribution, $N_{i}$ is the number of tokens within each type *i* out of the total number of types *K*, and ψ is the digamma function. The actual MI is then measured using:

2.6 $$MI=\hat{I}-{\hat{I}}_{sh},$$

where ${\hat{I}}_{sh}$ is the estimated lower bound of MI calculated from shuffled sequences, created by randomly permuting the concatenated sequences of individuals' songs.

**Figure 5.**
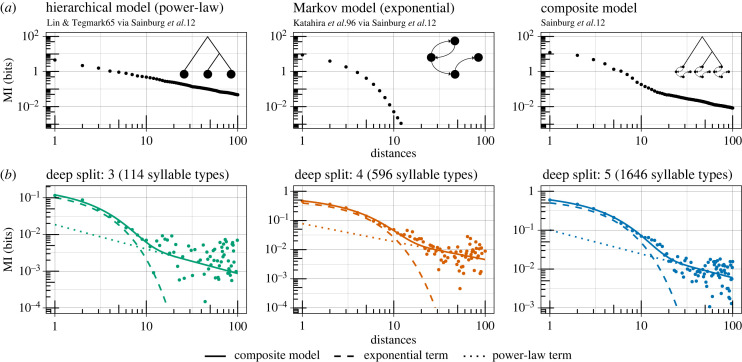
(*a*) Simulated mutual information decay curves from the hierarchical model (left) [65], the Markov model (centre) [99], and the composite model (right) [12] (data and inspiration for diagrams from [12]). (*b*) Computed mutual information decay curves for the observed data at three different levels of deep split. The solid line corresponds to the full composite model, while the dashed and dotted lines correspond to the exponential and power-law terms, respectively.

Sainburg *et al.* [12,66] simulated data from the hierarchical model of Lin & Tegmark [65], the Markov model of Katahira *et al.* [99] and their composite model in which Markov chains are nested within a larger hierarchical structure (figure 5).

To determine whether the observed MI decay was consistent with a Markov process, hierarchical process, or both, I fitted the following three decay models to the data:

2.7 $$\mathrm{exponential}\;\mathrm{decay}:\;y=ae^{-xb},$$

2.8 $$\mathrm{power-law}\;\mathrm{decay}:\;y=cx^{d},\;\mathrm{and}$$

2.9 $$\mathrm{composite}\;\mathrm{decay}:\;y=ae^{-xb}+cx^{d},$$

where *y* is the estimated MI and *x* is the distance between syllables [12,66]. Sainburg *et al.* [12] included an intercept *f* in all three models, but I removed it because it led to overfitting issues (e.g. exponential model with an extra ‘knee’ after the initial decay), did not significantly improve model fit (ΔWAIC<2), and was not included in Lin & Tegmark [65]. I focused my analysis on distances of up to 100 syllables to enable easy comparison with Sainburg *et al.* [12]. In the electronic supplementary material, I followed Sainburg *et al.* [12] in comparing the fit of each model at increasing distances from 100 to 1200 (the longest individual sequence is 1219), and found that the composite model outperforms both the exponential and power-law models at all distances.

At all three levels of granularity in syllable clustering, the composite model has a lower WAIC and higher $R^{2}$ than both the exponential and power-law models (see electronic supplementary material), suggesting that mutual information decay in house finch song is more consistent with a combination of Markovian and hierarchical processes. The composite model also outcompetes both the exponential and power-law models when fit to mutual information decay curves computed separately for each year of recording (1975, 2012 and 2019; see electronic supplementary material).

Interestingly, the transitions in the composite curves from figure 5 roughly correspond to the average length of a house finch song (approx. 12 syllables) [72]. I reran the analysis with individual song sequences rather than song bouts and found that the exponential model outcompeted the composite model at DS={3,4} (see electronic supplementary material). This suggests that individual song sequences may have Markovian structure within hierarchically organized song bouts.

## 3. Discussion

All three linguistic laws considered here are present in house finch song. Three out of the four measures of production cost, most importantly duration, are consistently and strongly negatively correlated with frequency, providing robust evidence for Zipf's law of abbreviation. Menzerath's law also found solid support, with a steeper negative relationship between song length and syllable duration than predicted by a model of production constraints [49]. Together, these results show clear evidence for efficiency—syllables that are difficult to produce are less common and more likely to appear in shorter songs. Mandelbrot's form of Zipf's rank–frequency law provided a good fit to the data and has a more convex shape than the original form, which is consistent with studies of other non-human communication systems that have more redundancy than human language [24–29]. I will limit my interpretation of this result, given ongoing debates about the cause of the rank–frequency law, but it is notable that the goodness-of-fit of the Zipf–Mandelbrot distribution to house finch song ($R^{2}>0.99$) is within the range of human language [21,91].

The two structural properties of language considered here are also found in house finch song. First, the syllable network has a small-world structure, characterized by high levels of clustering and low average path lengths, which is thought to reflect systematic structure and efficient recall in human language [52,53,100]. The small-worldness index of house finch song is within the range seen in humpback whales [24] and other songbirds at the first two levels of granularity in syllable clustering [26,56–59] (SWI∼1.69−4.7), but is much higher when syllables are over-split into types (SWI∼10−11). Second, the decay in mutual information between syllables with increasing distance has a similar shape to that found in several human languages and songbird species [12], which is associated with Markov chains nested within a larger hierarchical structure. Historically, birdsong sequences were assumed to follow simple Markov chains [99,101,102]. This result lends support to an emerging consensus that song bouts may be much more complex, containing long-range dependencies and hierarchical structures that resemble human language [11,12,103,104]. In combination, the small-worldness and mutual information decay of house finch song sequences suggest that they exhibit the kind of systematic structure that is thought to maximize expressivity while reducing learning costs [13,24,54], making it easier for more information to ‘pass through the bottleneck’ of social learning [1].

Notably, these patterns are consistent across three levels of granularity in syllable clustering. I have heard others studying the cultural evolution of birdsong refer to this as ‘fractal equivalency’—different resolutions of clustering should show similar forms of organization (D. C. Lahti 2020, personal communication). A practical conclusion of this finding is that information theoretic measures may not be reliable indicators of clustering quality in non-human communication systems [105].

A long-standing critique of Zipf's laws is that they may be statistical artifacts of other processes [106], starting with Miller's observation that randomly typing on keyboards can produce similar patterns [107]. That being said, random typing accounts are not realistic causal descriptions of how communication systems emerge, and there are good empirical reasons to doubt that they undermine efficiency accounts [21]. Randomly-generated texts produce rank-frequency distributions that differ from those in real corpora [108], random typing models are not truly neutral as they can be mathematically reframed as minimizing costs [32,109], and there is experimental evidence that both of Zipf's laws emerge from pressure for efficient communication [110,111]. In my view, the most important contribution of the random typing account is to highlight that the problem of equifinality—different processes leading to similar outcomes [112]—means that patterns resembling Zipf's laws are not sufficient to make conclusions about efficiency [20]. Multiple lines of evidence should be presented alongside other work demonstrating that efficiency is shaping the system (e.g. physical [73] and environmental [77] constraints), as I have done here. See Semple *et al*. [20] and Piantadosi [21] for more complete summaries of this debate.

Outside of linguistics, efficiency and complexity are often discussed in relation to cumulative cultural evolution (CCE). Definitions of CCE vary and a full review is outside of the scope of this study, but I will use the definition of Williams *et al.* [113]: “the accumulation of sequential changes within a single socially learned behavior that results in improved function”. Discussions of CCE often focus on increasing complexity over time [114], which was once thought to be a hallmark of human culture [115] but has now been observed in several non-human communication systems including humpback whale [116] and Savannah sparrow song [113]. Gruber *et al.* [1] make a convincing argument that efficiency deserves more attention in CCE, as increases in complexity in one domain require increases in efficiency in another. House finch song may be a good research model for how the interplay between efficiency and complexity drives CCE, as male house finches have a social learning bias for more complex syllables [9], possibly as an adaptation to female preferences for more complex songs [74–76], and there appears to be pressure for efficiency at the level of both syllables and songs. That being said, CCE may not be the best framework for understanding the interaction between efficiency and complexity in birdsong, as its logic is more difficult to apply to “aesthetic” behavior [117] especially when it is optimized for female preferences that evolve to maximize inclusive fitness rather than the specific properties of songs that males sing [118].

House finch song exhibits language-like efficiency and structure, but music-like structure has not been similarly studied in this species. In the last two decades researchers have identified aspects of birdsong, such as rhythm and pitch intervals in thrush nightingales [119,120], that closely resemble aspects of human music. Future studies should explore language- and music-like properties of birdsong in parallel across multiple levels of granularity to inform the ongoing debate about whether birdsong is more akin to music or language [121,122].

## Data Availability

The data and code for this study can be found in the Zenodo repository (https://doi.org/10.5281/zenodo.10689807) [123]. Supplementary material is available online [124].
